# Effect of ozone application on bovine carcasses in abattoir cold chambers

**DOI:** 10.1371/journal.pone.0321146

**Published:** 2025-05-02

**Authors:** Victoria Brusa, Viviana Restovich, Mariana Cap, Virginia Chiapparoli, Gabriela Grigioni, Leda Giannuzzi, Sergio Vaudagna, Gerardo Leotta

**Affiliations:** 1 IGEVET - Instituto de Genética Veterinaria “Ing. Fernando N. Dulout” (UNLP-CONICET LA PLATA), Facultad de Ciencias Veterinarias UNLP, La Plata, Argentina; 2 Frigorífico Arrebeef, Pérez Millan, Buenos Aires, Argentina; 3 INTA - Instituto Nacional de Tecnología Agropecuaria, Instituto Tecnología de Alimentos, Hurlingham, Argentina; 4 ICYTESAS – Instituto de Ciencia y Tecnología de Sistemas Alimentarios Sustentables, INTA-CONICET, Hurlingham, Argentina; 5 CIDCA - Centro de Investigación y Desarrollo en Criotecnología de Alimentos (UNLP-CONICET LA PLATA) La Plata, La Plata, Argentina; University of Jeddah, SAUDI ARABIA

## Abstract

Different technologies have been evaluated to promote food safety and improve the microbiological quality and shelf life of food. The aim of this work was to determine the effect of gaseous ozone on beef carcass weight loss and indicator microorganism counts in an exporting abattoir. Two gaseous ozone concentrations (3 and 10 ppm) were applied on carcasses from Experiment 1 (Exp. 1, n = 100 test, n = 100 control) and Experiment 2 (Exp. 2, n = 100 test, n = 100 control), respectively. Cold chamber without ozone was used as control in both experiments. For mesophilic aerobic organism (MAO), coliform and *Escherichia coli* counts, 100 cm^2^ of each carcass was swabbed before and 10, 20 and 30 h after ozone application. In Exp. 1, the carcass entire surface was swabbed for Shiga-toxin (*stx*) gene detection. The counts of MAO were influenced by treatment and sampling time in both experiments. In Exp. 1, control carcasses had higher counts than ozonated carcasses at all sampling times, whereas the opposite occurred in Exp. 2. Coliform count was affected by sampling time in Exp. 2, whereas *E. coli* count was not affected in any experiment. All samples analyzed were *stx*-negative. Differences in carcass weight loss were not significant. In conclusion, gaseous ozone was not effective to reduce bacterial load or carcass weight loss. To our knowledge, this is the first study evaluating ozone effect on beef carcasses conducted in a commercial abattoir, not at laboratory scale. Future research would help demonstrate whether the use of ozone impacts on the quality and sensory characteristics of beef.

## 1. Introduction

The food industry demands constant improvement in the quality of raw material. In recent years, different technologies have been evaluated to promote food safety and improve the microbiological quality and shelf life of food [1]. Beef is not only quite susceptible to microbial contamination, but also highly perishable due to intrinsic factors such as rich nutrients, high water activity and high pH [2,3]. Further, beef contamination with pathogenic and spoilage microorganisms can occur at different stages of the agri-food chain, from the abattoir to consumption [4].

Several antimicrobial treatments and intervention measures have been investigated to control the growth of spoilage and pathogenic microorganisms on beef products and extend their shelf life. They include antimicrobial physical treatments such as gamma irradiation [5,6], high hydrostatic pressure [7–9] and steam-vacuum system [10,11] as well as chemical interventions like hot water [12–17], organic acids (caprylic, lactic, peroxyacetic) [5,18–20], electrolytically generated hypochlorous acid and aqueous and gaseous ozone [21]. Of these, organic acids, hot water and irradiation have been approved by the Argentine health authority for their eventual application on beef [22,23]. The inclusion of decontamination methods and intervention measures in Hazard Analysis and Critical Control Point (HACCP) programs has helped to achieve significant reductions in pathogenic bacteria and/or extend the shelf life of beef carcasses [24].

Ozone has been granted the generally recognized as safe (GRAS) status for direct food contact and approved for application in the treatment, storage and processing of foods, including meat and poultry [25]. Ozone instantly decomposes to oxygen in air and water [21,26] and can be used in the aqueous or gaseous phase. These characteristics confer ozone a high oxidative power and rapid decomposition, making it effective against bacteria, viruses, fungi and mycotoxins, without leaving any toxic by-products or residues [26–28]. In addition to these properties, consumers´ acceptance makes it an attractive alternative for application in the beef industry [29,30]. Temperature, pressure, and relative humidity appear to be the main ambient factors affecting gaseous ozonation. Other factors such as the properties of the material to be decontaminated, the microorganism, the method of substrate contamination and ozone generation and the exposure dosage also affect the efficiency of the ozonation process [26].

Different reports have evaluated the ability of gaseous ozone to reduce beef carcass weight loss, drop the load of pathogenic and spoilage microorganisms, and extend beef shelf-life [21,31]. Ozone application not only allows obtaining more tender meat, but also significantly decreases carcass weight loss, stabilizes pH, improves the visual appearance of the product, and suppresses unpleasant odors inside cold chambers [32].

The effectiveness of gaseous ozone on the microbial load and shelf life of meat has also been investigated [1,21]. Laboratory scale studies have tested gaseous ozone at different concentrations (0.44–1000 ppm) and application times (minutes-days) on beef, broiler, pork and turkey, showing variable results [33–36]. To our knowledge, the effect of ozone on beef at commercial abattoir scale has not been determined thus far.

The aim of this work was to evaluate the effect of gaseous ozone on beef carcass weight loss and indicator microorganism counts in an exporting abattoir.

## 2. Materials and methods

**Fig 1:**
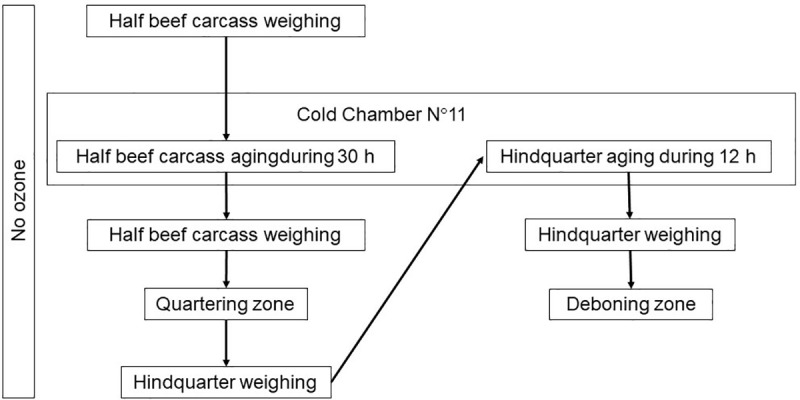
Sequence of processes in non-ozonated beef products.

**Fig 2:**
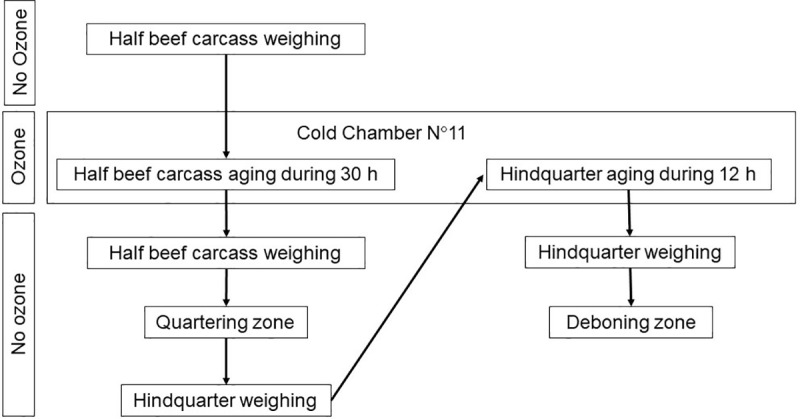
Sequence of processes in gaseous ozone-treated beef products.

The study was carried out in an abattoir located in the province of Buenos Aires, Argentina. The abattoir produces beef for export and for local markets and it applies HACCP, Good Manufacturing Practices (GMP) and Good Hygiene Practices (GHP). Sampling was approved by the National Service of Agrifood Health and Quality of Argentina (SENASA, for its Spanish acronym). The study did not require approval by the ethics committee of the authors’ institutions. It was divided into experiment 1 (Exp. 1, conducted in July 2021) and experiment 2 (Exp. 2, conducted in September 2021) according to the gaseous ozone concentration applied (3 and 10 ppm, respectively). A gaseous ozone generator producing 60 kg ozone per hour was used (OZONA S.R.L, Villa Marteli, Buenos Aires, Argentina). Each experiment was conducted on separate cold chambers, whose characteristics are detailed in Table 1. Information regarding ambient ozone concentration, room temperature and relative humidity of each cold chamber, carcass temperature and pH is detailed in Table 2. During both experiments, environmental and carcass variables and the process flow corresponded to the routine working conditions of an abattoir. The sequence of processes to which the carcasses and hindquarters were submitted is presented in Figs 1 and 2. A self-contained breathing apparatus with low pressure and air demand circuit was used to enter into cold chamber N°12 (test), (MSA, Don Torcuato, Buenos Aires, Argentina).

**Table 1:** Cold chamber characteristics and gaseous ozone concentrations used in experiments 1 and 2.

Cold Chamber	Dimension (m)	Area (m^2^)	Volume (m^3^)	Capacity (carcasses)	Gaseous ozone^*^
Control	14.85 x 4.1 x 5.2	60.8	316.2	100	Experiments 1 and 2: no ozone applied
Test	14.85 x 4.5 x 5.2	68.8	347.4	100	Experiment 1: 3 ppm (6 mg O_3_/m^3^) Experiment 2: 10 ppm (21 mg O_3_/m^3^)

* Maximum concentration reached at each experiment.

**Table 2:** Measured variables, measurement times and measuring instruments used in the experiments.

Variable	Measured value (min-max)		Time	Instrument
	Control chamber	Test chamber		
Ambient ozone concentration (ppm)			every one hour	QB2000N/T Fixed Gas Module Monitor Transmitter (Henan Chicheng Electric Co., Ltd., Henan, China).
Exp. 1				
T1		2–6		
T2		2–4		
T3		2–4		
Quartering		2–4		
Exp. 2				
T1		8–12		
T2		9–11		
T3		8–12		
Quartering		8–15		
Room temperature (°C)			every 5 min	Data logger HOBO MX2300 (ONSET, Bourne, Massachusetts, United States).
Exp. 1				
T0	3.98–18.3	2.24–19.85		
T1	4.28–5.38	2.16–3.34		
T2	−1.47–4.86	−1.62–3.26		
T3	−3.47–3.39	−2.81–2.07		
Quartering	−3.61–1.24	−0.32–7		
Exp. 2				
T0	7.33–18.66	9.82–19.53		
T1	5.23–8.46	3.13–12.38		
T2	4.47–6.22	2.28–4.97		
T3	0.91–5.02	0.39–3.72		
Quartering	−2.3–8.06	−2.18–5.47		
Relative humidity (%)				
Exp. 1				
T0	40.14–92.96	44.39–88.99		
T1	73.09–95.1	75.65–91.19		
T2	62.68–91.62	72.67–92.52		
T3	62.62–98.64	70.96–96.10		
Quartering	66.04–99.16	90.05–97.61		
Exp. 2				
T0	47.08–84.1	58.73–92.96		
T1	51.53–81.43	59.33–86.64		
T2	59.71–86.74	63.7–83.75		
T3	59.82–91.72	67.67–88.65		
Quartering	58.89–100.0	67.01–96.56		
Carcass temperature (°C)			T0, T1, T2 and T3	Meat thermometer (HANNA, CABA, Argentina), range from -50–150°C with a resistance thermotype sensor, accuracy of ± 0.3 ºC.
Exp. 1				
T0	13.9–20.3	10.4–16		
T1	10.3–13.9	6.3–10.4		
T2	7–10.7	4.7–6.8		
T3	3.8–5.7	1.7–2.5		
Exp. 2				
T0	20.1–30.7	26.9–30.3		
T1	10.3–12.4	6.0–7.9		
T2	5.0–8.0	3.0–5.3		
T3	5.7–8.8	3.2–4.1		
Carcass pH^1^			T3 (at the end of carcass aging stage)	Digital instrument (HANNA brand puncture electrode), FC 202D Din connection electrode, reading range from 0 to 12 pH and 0–50°C.
Exp.1	5.1–5.79	5.4–5.75		
Exp. 2	5.3–5.78	5.22–5.76		

T0, T1, T2 and T3: sampling times before ozone application and 10 h, 20 h and 30 h after ozone application, respectively.

^1^Measured at the external side of the dorsal muscle, between the 12th and 13th rib. For pH measuring instruments, the company defines that the maximum difference between the buffer value and that indicated by the instrument must be at most ± 0.05.

### 2.1. Sample collection

Microbiological carcass samples from Exp. 1 and 2 (n = 200 each) were obtained using a sterile sponge (Whirl-Pak Speci-Sponge, Nasco, USA) soaked in 10 ml buffered peptone water (BPW) (Acumedia Manufacturers, Lansing, MI). In each experiment, one sponge was used for indicator microorganism count by swabbing four carcass areas of 100 cm^2^ each (chest, neck, buttock and posterior lateral hock). First, the chest and neck area were swabbed with one side of the sponge (ten strokes in two directions, from left to right and from top to bottom). Then, the same sponge was flipped to the other side to swab the buttock and posterior lateral hock as aforementioned. Samples were taken before (T0, n = 25) and 10 h (T1, n = 25), 20 h (T2, n = 25) and 30 h (T3, n = 25) after ozone application. At each time point, a different group of carcasses was sampled. In Exp. 1, a second sterile sponge was used to swab the carcass entire surface (external and internal side) for Shiga-toxin (*stx*) detection at T0 (n = 25) and T3 (n = 25) (Table 3).

**Table 3:** Samples taken and sampling times.

Sampling	Carcass ID	*stx* ^1^	MAO and C/EC
Time			
T0	1–25	E1	E1-E2
T1	26–50		E1-E2
T2	51–75		E1-E2
T3	76–100	E1	E1-E2
Surfaces			
400 cm^2^			x
All beef carcass^2^		x	
N =^3^		100	400

ID: identification; E1: experiment 1, E2: experiment 2;

^1^*stx* detection;

^2^internal and external;

^3^total analyses performed; MAO: mesophilic aerobic organisms; C/EC: Coliforms/*E. coli*

To evaluate weight loss, carcasses were weighed before being placed in the cold chamber using a slaughter weighing scale model LT 600E, 1000 kg load cell up to 500 g tolerance), and before quartering using a quarter weighing scale model LT 600E, 1000 kg load cell up to 100 g tolerance. A quarter weighing scale was also used to weigh hindquarters before placing them in the cold chamber and before deboning.

### 2.2. Bacteriological analysis

Carcass samples were analyzed for mesophilic aerobic organisms (MAO), coliforms and *E. coli* with 3M™ Petrifilm™ aerobic count plates (3M™, Minnesota, USA) and 3M™ Petrifilm™ *E. coli*/coliform count plates (3M™). After placing samples in a stomacher bag, 15 ml of BPW was added. After mixing for 30 s, 1 ml of sample was placed into each Petrifilm plate, incubated and counted according to the manufacturer’s specifications [37,38]. Results were expressed as log CFU/cm^2^. *stx* detection was performed using BAX® System Real-Time PCR Assay for Shiga toxin-producing *E. coli* (STEC) screening (DuPont Corporation, DE, USA) according to the manufacturer’s instructions [39].

### 2.3. Statistical analysis

The effect of each ozone concentration on carcass weight loss was evaluated using two-tailed Student’s *t*-test for independent variables. The effect of each ozone concentration on microbial load was evaluated using a generalized linear model with gamma distribution and logarithmic link function because the frequency distribution of the dependent variable (indicator microorganism count on carcasses) was skewed to the right (neither normal nor homoscedastic). In Exp. 1, the fixed factors were treatment (3 ppm ozone/no ozone) and sampling time (T0 to T3), and the dependent variables were MAO, coliforms and *E. coli*, as appropriate. In Exp. 2, the fixed factors were treatment (10 ppm ozone/no ozone) and sampling time (T0 to T3), and the dependent variables were MAO, coliforms and *E. coli*, as appropriate. All statistical analyses were performed using InfoStat (Universidad Nacional de Córdoba). Significance threshold was set at *p *< 0.05.

## 3. Results

### 3.1. Indicator microorganism count

The counting of MAO was influenced by treatment and sampling time in both experiments. In this sense, MAO counts increased as time progressed in both experiments (Tables 5 and 6, S1 Table Exp. 1 and S2 Table Exp. 2). In Exp. 1, control carcasses had higher MAO counts than ozonated carcasses at all sampling times (Table 5, S1 Table Exp. 1). Instead, ozonated carcasses in Exp. 2 had higher MAO counts than control carcasses at all sampling times (Table 6, S2 Table Exp. 2). In Exp. 1, coliform counts were higher at T3 than at previous sampling times in both chambers (Table 5, S1 Table Exp. 1). In Exp. 2, coliform counts were affected by sampling time (*p *= 0.013), and the initial coliform count decreased in subsequent sampling times in both chambers (Table 6, S2 Table Exp. 2). Also, a treatment x sampling time interaction was observed in Exp. 2 (*p* = 0.013) with respect to coliform counts (Table 4). In Exp. 1, ozone application and sampling time had no significant effects on coliform (*p* = 0.738; *p* = 0.448) and *E. coli* (*p* = 0.685; *p* = 0.053) counts (Table 4). Also, treatment and sampling time did not interact with MAO (*p* = 0.06), coliform (*p* = 0.742) and *E. coli* (*p* = 0.924) counts. Regarding Exp. 2., treatment did not significantly affect coliform (*p* = 0.06) and *E. coli* (*p* = 0.245) counts, and sampling time did not affect *E. coli* counts (*p* = 0.255). Treatment x sampling time interaction with MAO (*p* = 0.772) and *E. coli* (*p* = 0.285) counts was not observed. The results of MAO and *E. coli* counts were within the established quality control standards [40–42], while there are not established quality control standards for coliform counts. Means and marginal means of MAO, coliform and *E. coli* counts on beef carcasses from each cold chamber are shown in Tables 5 and 6.

**Table 4:** Statistical results of indicator microorganism counts. The fixed factors were treatment and sampling time and the dependent variables were MAO, coliforms and *E. coli.*

	Factor				Factor Interaction	
	Treatment		Sampling time			
Experiment	1	2	1	2	1	2
MAO	< 0.001	0.001	0.035	< 0.001	0.06	0.772
Coliforms	0.738	0.06	0.448	0.013	0.742	0.013
*E. coli*	0.685	0.245	0.053	0.255	0.924	0.285

**Table 5:** Means and marginal means of mesophilic aerobic organism, coliform and *E. coli* counts on beef carcasses from each cold chamber in Experiment 1.

Sampling Time	Mesophilic aerobic organisms^*^			Coliforms^*^			*E. coli* ^*^		
	Cold chamber		Marginal mean	Cold chamber		Marginal mean	Cold chamber		Marginal mean
	Control	Test		Control	Test		Control	Test	
T0	3.79	3.44	3.6 ^a,b^	1.44	1.50	1.47^a^	1.33	1.35	1.34^a^
T1	3.59	3.57	3.58^a^	1.45	1.30	1.38^a^	1.30	1.30	1.30^a^
T2	3.73	3.65	3.69^a,b^	1.30	1.40	1.35^a^	1.30	1.30	1.30^a^
T3	3.87	3.62	3.74^b^	1.46	1.56	1.51^a^	1.30	1.30	1.30^a^
Marginal mean	3.74	3.57		1.42	1.44		1.31	1.31	

* log CFU/400 cm^2^; ^a,b,c^ For each microorganism, different letters indicate statistically significant differences.

**Table 6:** Means and marginal means of mesophilic aerobic organism, coliform and *E. coli* counts on beef carcasses from each cold chamber in Experiment 2.

Sampling Time	Mesophilic aerobic organisms^*^			Coliforms^*^			*E. coli* ^*^		
	Cold chamber		Marginal mean	Cold chamber		Marginal mean	Cold chamber		Marginal mean
	Control	Test		Control	Test		Control	Test	
T0	3.49	3.64	3.56^a^	1.30	1.46	1.38^a^	1.44	2.66	2.05^a^
T1	3.50	3.69	3.60^a^	1.30	1.30	1.30^b^	1.30	1.38	1.34^a^
T2	3.85	3.98	3.91^b^	1.30	1.30	1.30^b^	1.3	1.42	1.36^a^
T3	3.79	3.86	3.82^c^	1.30	1.30	1.30^b^	1.30	1.30	1.30^a^
Marginal mean	3.66	3.79		1.30	1.34		1.34	1.69	

* log CFU/400 cm^2^; ^a,b,c^For each microorganism, different letters indicate statistically significant differences.

### 3.2. *stx* detection

All samples analyzed were *stx*-negative.

### 3.3. Weight loss

Comparison of tested samples from Exp. 1 and Exp. 2 did not result in significant differences with reference to controls (Exp. 1: carcass, *p* = 0.626; hindquarter, *p* = 0.476; Exp. 2: carcass, *p* = 0.103; hindquarter, *p *= 0.289) (Table 7, S1 Table Exp. 1 and S2 Table Exp. 2).

**Table 7:** Carcass and hindquarter weight loss results.

		Experiment 1		Experiment 2	
		Control	Test	Control	Test
Carcass					
$\overset{―}{X}$ (range) weight (kg)^*^	T0	147 (125–164)	133 (111–166)	128 (96–157)	126 (96–126)
	Before quartering	144 (122–161)	130 (109–163)	126 (95–155)	124 (94–179)
Weight loss (%)^*^		1.9	1.7	1.2	1.0
*p* =		0.626		0.103	
Hindquarters					
$\overset{―}{X}$ (range) weight (kg)^*^	After quartering	59 (50–73)	57 (46–73)	55 (43–68)	52 (39–79)
	Before deboning				
Weight loss (%)^*^		2.5%	1.1%	0.4	0.8
*p* =		0.476		0.289	

* Calculated considering the weight of all carcasses aging in the same cold chamber.

## 4. Discussion

Improving carcass performance and the microbiological quality and sensory characteristics of beef is a common goal of the beef industry. The Ministry of Agriculture, Livestock and Fisheries of Argentina recommends the application of low gaseous ozone concentrations in atmospheres where beef is processed and stored to achieve significant reductions in weight losses, ensure food safety, improve sensory quality (more tender beef, better visual appearance) and extend shelf life [32]. In this sense, results of scientific publications evaluating the effect of gaseous ozone on meat under controlled conditions are in agreement with such recommendations [21,33,34,43]. Although these reports set high expectations for the use of gaseous ozone as an alternative to improve the cost efficiency of the Argentine beef industry, there are no studies at abattoir scale demonstrating such benefits. The current study was aimed at verifying the effectiveness of gaseous ozone on beef at abattoir scale.

Few scientific papers have evaluated the effectiveness of ozone in reducing meat weight loss [33,43], and only one has estimated this parameter in bovine carcasses [44]. Lab-scale studies have reported drip loss reduction in poultry meat treated with ozone [43]. Another study has reported improved water holding capacity of skinless turkey breast slice pieces exposed to gaseous ozone (5 ppm) in a 14 L glass chamber during 6–8 h at 22 °C [33]. In the present study of bovine carcasses and forequarters performed at an exporting abattoir, gaseous ozone did not induce significant weight loss reductions in any experiment. To our knowledge, only one study has determined the effect of 0.03 ppm ozone on beef carcasses at an experimental abattoir after chilling carcasses for 24 h at 10 °C before randomly assigning alternate sides to the ozone or control group and evaluating them following 9 days of aging [44]. Unlike our results, weight loss was significantly higher in ozonated carcasses compared to control sides, suggesting greater evaporative weight losses in ozone-treated than control sides. In our study, the conditions and ripening times used in exporting abattoirs were applied. Probably, longer ozone exposure of bovine carcasses and forequarters would have produced similar results to those reported by Greer and Jones [44] (Table 2).

Considering that meat is a highly perishable food, reducing the microbial load is important to extend its shelf life. Gaseous ozone has been proposed as an effective alternative to reduce foodstuff microbial load, including meat [21]. Some studies have evaluated its effect on naturally contaminated beef or beef experimentally inoculated with pathogenic and non-pathogenic microorganisms. In a laboratory-scale study, the application of 72 ppm gaseous ozone for 24 h reduced *E. coli* (0.7 log_10_ cycles) and total aerobic mesophilic heterotrophic microorganism counts (2.0 log_10_ cycles) from beef samples (0.5 cm thick and 6.0 cm in diameter) [34]. In the current study, gaseous ozone was not effective for this purpose. Significant differences in MAO and coliform counts in favor of non-ozonated chambers were identified. Since experiments were carried out in a commercial abattoir, these results would be explained by differences in initial natural bacterial loads between carcasses. Other laboratory scale studies in beef portions (0.3 cm thick and 6 cm in diameter discs) and minced meat (20 g) reported similar reductions in microbial counts to those found by Coll Cárdenas *et al*. [34] and significant reductions in the load of *E. coli* O157 and *Listeria monocytogenes* [31,45]. In the present study, the application of gaseous ozone had no antimicrobial effect on carcasses from a commercial abattoir. Stratakos and Grant [46] exposed 5 × 5 cm *E. coli*-inoculated beef portions to 3400 and 15000 ppm gaseous ozone for 5 min. These authors reported that the ozone treatments did not have a significant antimicrobial effect against *E. coli* either immediately after treatment or during storage, which is in coincidence with our results. Once again, to our knowledge the only study evaluating the antimicrobial effect of gaseous ozone on beef at a similar scale was performed by Greer and Jones [44] in an experimental abattoir. The mentioned authors applied 0.03 ppm gaseous ozone on beef carcasses during 9 days of aging and reported about 10-fold greater bacterial numbers on control carcasses. However, the inhibition of bacterial growth on carcass surfaces could be attributed to significantly greater evaporative losses from ozonated-treated carcasses, which limited bacterial growth, and cannot be unequivocally attributed to an antimicrobial ozone effect. Since Greer and Jones [44] performed the study under the extensive hygienic conditions of a research abattoir, they recommended confirming the results under commercial conditions, where the levels of bacterial contamination would be more realistic. The present study was performed under realistic levels of bacterial contamination, without finding statistically significant differences in antimicrobial ozone effect on beef carcasses.

Laboratory scale assays have evaluated different gaseous ozone concentrations and exposure times to reduce microbial load (mainly experimentally inoculated) in poultry, pork and beef [33,47]. Even though the high ozone concentrations applied reduced the microbial load, they also produced unacceptable alterations in meat (darkening and drying of the exposed muscle surfaces, lipid oxidation, reduced size of loin eye) [31,33,34]. Therefore, the benefits of the antimicrobial effect of ozone would be possibly offset by the negative consequences promoted by its application in those conditions [44]. Although gaseous ozone did not have antimicrobial effect on beef under the conditions currently studied, it would be interesting to evaluate its effect on the sensory parameters and shelf life of refrigerated/frozen vacuum-packaged meat.

Shiga toxin-producing *Escherichia coli* (STEC) are considered a hazard in beef abattoirs applying HACCP plans [48]. In Argentina, several studies have evaluated the effectiveness of different antimicrobial methods to decrease STEC from beef. Furthermore, beef abattoirs implemented intervention measures and improvement actions to reduce the presence of STEC on beef and abattoir facilities [18,49,50]. Following previous research, we aimed to evaluate the effectiveness of gaseous ozone to eliminate *stx* from beef carcasses in a commercial abattoir. However, since the actions implemented to reduce STEC in abattoirs were effective, all the samples analyzed were negative for *stx*. Therefore, the gaseous ozone effect against STEC could not be determined.

The results obtained in studies using controlled laboratory conditions are disparate and do not allow reaching unequivocal conclusions regarding the effect of ozone on meat. In this study, gaseous ozone was not effective in reducing the load of indicator microorganisms or weight loss in commercial abattoir carcasses at the concentrations evaluated. In this context, several issues should be considered before choosing gaseous ozone application in a commercial abattoir: a) clearly establishing the objectives for the application (for example, improvement of beef quality attributes); b) evaluating the application conditions (ozone concentration, exposure time, presence of organic matter, temperature, pressure and relative humidity, among others); c) considering the levels of ozone exposure in the workplace environment and the use of appropriate personal protective equipment; and d) evaluating the cost associated with equipments and their installation.

## 5. Conclusion

We conclude that the application of 3 and 10 ppm gaseous ozone in cold chambers was not effective for beef carcass weight loss or bacterial load reduction on half-carcasses. Nevertheless, future research would help demonstrate whether the use of ozone impacts on the sensory quality of beef.

## Supporting information

S1 Table Data tables from Experiment 1. (XLSX)

S2 Table Data tables from Experiment 2. (XLSX)
